# Controlling Reproduction and Disrupting Family Formation: California Women’s Prisons and the Violent Legacy of Eugenics

**DOI:** 10.3390/soc14050073

**Published:** 2024-05-19

**Authors:** Vrindavani Avila, Jennifer Elyse James

**Affiliations:** 1 Cultural Studies Graduate Group, University of California, Davis, CA 95616, USA; 2 Institute for Health and Aging, School of Nursing, University of California, San Francisco, CA 94158, USA

**Keywords:** incarceration, eugenics, racial capitalism, state violence

## Abstract

Prisons in the United States serve as a site and embodiment of gendered and racialized state violence. The US incarcerates more people than any other nation in both numbers and per capita rates. Individuals incarcerated in women’s prisons are 10% of the total prison population, yet women’s prisons remain understudied, and the violence that occurs in women’s facilities is rampant, widespread, and operates in particular racialized and gendered ways. This paper centers the forced sterilizations that occurred in California state prisons over the last two decades. We consider how reproduction and the nuclear family have served as a primary site of racial capitalism and eugenic ideology. While eugenic policies were popularized and promoted across the US and globally in the 20th century, the violent ideas underlying eugenic ideology have been a constant presence throughout US history. The height of the eugenics era is marked by the forcible sterilization of institutionalized ‘deviant’ bodies. While discussions of eugenics often center these programs, the reach of eugenic policies extends far beyond surgical interventions. We utilize a reproductive justice lens to argue that the hierarchical, racialized social stratification necessary for the existence of prisons constructs and sustains the ‘deviant’ bodies and families that predicate eugenic logic, policies, and practices. In this conceptual paper, we draw from ongoing research to argue that prisons, as institutions and as a product of racial capitalism, perpetuate the ongoing violent legacy of eugenics and name *abolition* as a central component of the fight to end reproductive oppression.

## 1. Introduction

### 1.1. Moonbeam’s Story

Moonbeam is a Native American woman in her 50s. She lives at home with her parents in California, where she helps to care for her mom who has dementia. Moonbeam moved home recently after she was released from a state prison in California, where she was incarcerated for more than 25 years.

While Moonbeam was incarcerated, she went to see a gynecologist for a routine pap smear. She was not experiencing any symptoms or problems, but her physician recommended some additional scans. She agreed and learned she had two growths, which her doctor said could potentially turn into cancer. He recommended surgery and she agreed.

Moonbeam went to the hospital for the procedure and was handed a consent form to sign. No one reviewed it or went over the procedure with her. Reflecting on the consent process, she recalled, “When I agreed to the procedure, I agreed to them removing the growths, but I never read the paperwork because I’m thinking this is a doctor and he works for the state of California, so they hired somebody legit. Well, he wasn’t. He wasn’t.”

After the surgery, as she was recovering, Moonbeam recalled sudden and extreme symptoms. “Soaked in my neck”, she said, “it’s between my breasts, it’s just dripping down my face. I’m in puddles of sweat.” She couldn’t figure out what was going on and assumed it had something to do with this surgery. A few days later, she had an appointment in the medical unit at the prison to have her dressings changed.

She asked the nurse who came in, “What exactly was done? What kind of procedure was done because this sweating is like way too much, you know? I’m young, like I don’t know what’s going on with my body.” The nurse responds to her quite casually, saying, “oh, well, you had a full hysterectomy.”

Moonbeam had no idea.

A few days later, she saw the doctor—the same one who recommended the scan and the surgery to remove these growths. She described questioning him, saying, “You said I had two growths…you took everything out?” She described his response, the emotion of that time thick in her voice:

He sat back like very smug in the chair and looked at me. He said, ‘let me tell you something… I’m tired of you pretty girls coming to prison, you get out… you have sex with God knows whoever and you come back to prison and you’re pregnant, you have these babies that end up in the system and we have to pay taxes for them.’ And my mouth just hit the floor.

In remembering her response at the time, Moonbeam said, “it came to me. I was like this man just played God with my life. He took the choice whether I could have kids or not away from me without even asking. And so, for a long time I walked around thinking, damn, this guy got away with this. And like I’m in the system. What can I do?”

### 1.2. Background

We interviewed Moonbeam as a part of an ongoing research project on the forced sterilizations performed on incarcerated people in California in the 21st century. In 2013, the Center for Investigative Reporting (CIR) issued a bombshell report asserting that at least 144 women incarcerated in California state prisons were sterilized via tubal ligation without proper informed consent between 2005 and 2012. This occurred at a time when both the practice of compulsory sterilization in state facilities had been outlawed [1] and the state prison system had declared that tubal ligations were not “medically necessary” and thus should not be a covered expense for incarcerated patients [2]. Dr. James Heinrich, an ObGyn working inside California prisons, when asked by Corey Johnson for the CIR article about the $147,460 of taxpayer funds spent on sterilization procedures, described the cost as minimal “compared to what you save in welfare paying for these unwanted children—as they procreated more” [2]. This quote highlights the eugenic logic held by at least one physician within this system and implies that these sterilizations were in fact an extension of the far-reaching California eugenics program—a eugenic legacy many Americans thought had stopped more than half a century prior.

The term eugenics was coined in 1883 by Francis Galton and described the theory, rationale, and methods for organizing the population according to Eurocentric ideals of ‘superior’ human characteristics, bodies, and behaviors [3]. Eugenic logic was popularized, within the sciences and to the general public, through defining, legitimizing, and legalizing an ideal mind/body of a citizen—often those who were considered white, able-bodied, cisheterosexual, and economically stable. Eugenics programs across the US were a mainstay, promoted throughout state public health initiatives [4] as a tool to improve society’s “mental and physical health”, while in actuality, “eugenics enforced social judgements about race, class, and gender cloaked in scientific terms” [5]. While the methods of, approaches to, and discourses surrounding eugenics in the 19th and 20th centuries focused on controlling reproduction through surgical procedures, driven by the idea of desirable and undesirable and heritable traits, the core tenets and logics were not new but were central features of enforcing social hierarchies throughout US history.

The height of the eugenics era in the 20th century is marked by the forcible sterilization of, often institutionalized, “deviant” bodies. The rationale for the continued legality of compulsory sterilizations was, at least in part, rooted in a desire to disrupt criminality before it occurred by preventing “born criminals from bearing more of their kind” [6]. As Justice Oliver Wendell Holmes wrote in his infamous opinion for *Buck v. Bell*, “It is better for all the world if, instead of waiting to execute degenerate offspring for crime or to let them starve for their imbecility, society can prevent those who are manifestly unfit from continuing their kind” [7].

More than 60,000 people in the US were sterilized under state eugenics programs in the 20th century, with California having the most far-reaching program [8]. An estimated 20,000 people were sterilized in state hospitals and institutions in the 20th century [3], with the practice not formally ending until 1979. While the popularity of compulsory sterilization waned in the 1950s, eugenic logic evolved and continued to live on in various institutions [9]. Ideas of segregation, institutionalization, detention, and the incentivizing or disincentivizing of reproduction have remained constant [10]. Abortion politics, welfare policies, and anti-immigrant discourses all tell the tale of the weaponization of the perceived threat of undesirable, disabled, and deviant (i.e., Black, Brown, “hyper sexualized”, and poor) children and the need to further the reproductive increase of the ideal (i.e., white, middle class, normative) family. State and federal policies such as segregation in the 1950s and 1960s, the “welfare queen” stereotype, which was used to justify the forced implantation of Norplant, Temporary Assistance for Needy Families (TANF) reform in the 1990s, and discourses such as “anchor babies” in the 2010s [4,11–13] all make clear that the reproduction of the ideal family is utilized as a violent tool of eugenics to contain and manage certain populations.

While the nuclear biological family is a structure presumed to be natural, normal, or innate, the nuclear family is based in a specific historical, temporal, and spatial arrangement and is used to uphold particular racialized and gendered formations and delegitimize social arrangements that do not align with or support the dominant structure. Eugenic practices, policies, and processes leaned heavily on the concept of the nuclear family to push and cement categories of belonging, protection, safety, and legalization that further alienated and ultimately justified violent othering through law and policy [3].

In this paper, we utilize racial capitalism to trace how the state produces and reproduces violence and control through reproductive oppression and the weaponization of an ideal nuclear family. We began with a story of forced sterilization within a California prison and extend our analysis to understand how prisons and incarceration are sites of eugenic practice and violent reproductive oppression. While US eugenics era laws and programs argued for forced sterilization and other eugenic practices in the name of “public health” [4], we argue that a central form of work of the US system of incarceration has been to construct “deviant” bodies as dangerous and allow for the perpetuation of state violence against them for the purposes of “public safety”. We consider not only how healthcare providers used forced sterilizations to enact violence and harm on patients under their care but consider what it means to incarcerate people for decades at a time, through their reproductive years, limiting or eliminating their reproductive capacity and disrupting family formations. We argue that the practice of incarceration serves as a form of *structural eugenics* and extends a long history of violent control of reproduction and family formation by the state.

This paper draws on two years of community-based, multi-method, critical qualitative analysis focused on the forced sterilizations that were performed on people incarcerated in California’s women’s prisons. We conducted 40 interviews with patients, providers, and experts in law, medicine, and reproductive justice, in an effort to understand how forced and coercive sterilizations occurred and if and how we can protect the rights and autonomy of people currently incarcerated. From January 2022 through December 2023, we followed the implementation of the California Forced or Involuntary Sterilization Compensation Program. Observing the implementation of this program, which provided compensation for survivors of forced sterilization in state institutions, allowed us to understand how the state views consent, medical necessity, and eugenics. While we draw on our research, this paper is focused on our conceptual understandings of eugenics, shaped by the insights of survivors and advocates, rather than an empirical paper based on research findings. We share the words and insights of a few of our participants not to analyze them as data but to demonstrate the ways in which we have theorized alongside them and learned from their lived experience of the modern enactment of racial capitalism and eugenic logic.

## 2. Racial Capitalism and the Creation of the Other

“We are a subset, you know? We are a subset of the human race. And we’ve seen them do it to Black and Brown women in the free world… that’s just the order of things, right? In their minds, we’re subhuman, right? We’re less.”

—Formerly Incarcerated Survivor

Racial capitalism is a theoretical framework describing how hierarchical social formations, specifically those based on racialization, are foundational and embedded in the structure and site of capitalist accumulation. In his foundational work on the topic, Cedric Robinson provides a historical materialist analysis of how race was first constructed and produced throughout Europe to justify violent dispossession under feudalism. Racial capitalism describes how various processes of accumulation require “loss, disposability, and the unequal differentiation of human value, and racism enshrines the inequalities that capitalism requires” [14]. These same principles were extended into the US through colonization and the transatlantic slave trade, establishing and cementing social political systems that aimed to promote capitalist accumulation while protecting white, normative ideals of family [15].

While the mechanisms of dispossession have changed over time, the framework of capitalism’s reliance on and creation of inequality has remained a constant feature of the US capitalist system. Much as the survivor we quoted described, racial capitalism relies on a central logic of categorizing people and establishing an *other* based on a differential hierarchical framework. That is, capitalism itself needs violent modes of othering such as racism, misogyny, transphobia, ableism, and other forms of oppression to sustain itself [16–18].

This othering structure has been reproduced time and time again, with eugenics taking up this violent ideology as both a mechanism and an ideological approach for enacting racial capitalism. Eugenics became a naturalized and institutionalized practice in the 19th and 20th century, where the ideological apparatuses of the state, such as state public health initiatives, determined who was “fit” to reproduce and who was not, instilling a hierarchical system of value based on subjective white supremacist social formations. The categorization necessary for violent dispossession under racial capitalism creates the possibility of eugenic logics, which in turn creates the mechanisms for forced family disruptions and redefinitions. Racial capitalism relies on hierarchical social formations to maintain itself, and violence through policing, punishment, and confinement is a central component to sustaining its structure. Eugenics is a key iteration of policing and management that has been institutionalized and grounded in policies and law. Managing and controlling reproduction has always been of importance to the racial capitalist regime, as reproductive capacities dictated issues of labor and capital accumulation. Eugenic logic became a credible practice, seemingly tied to the inner workings of racial capitalism, suggesting that “eugenics is the love language of [racial] capitalism” [19].

## 3. Eugenics as the Love Language of Racial Capitalism: Examples across History

“Listen, we are property. We are property once we are in there and that’s what you’re told is to shut up and just do what’s to be done, right? … You get into prison you are their property.”

—Formerly Incarcerated Survivor

While eugenics as a named ideology was promoted and popularized in the 20th century, the violent ideas underlying the movement have been a constant unnamed presence throughout the development of racial capitalism. From the earliest days of the colonial period, we can see how the laws and practices that justified the treatment and violent dispossession of African and Indigenous people were about determining the value of who is deserving to reproduce biologically and socially. Eugenics as an ideology, as a practice, is innate to racial capitalism as it materializes and legitimates constructed ideas of the other and justifies violent treatment.

The idea of a biological nuclear family is rooted in colonial ideals and is embedded in US laws, constructs various institutions, and polices social structures that fall outside this formation [20]. The biological nuclear family is tied to formations of slavery, property, and wealth, where family is established as a protected site, through biological reproduction, to enable the accumulation of wealth, the stabilization of borders and boundaries, and the policing of social and institutional norms [21]. The biological nuclear family serves as an ideological state apparatus that enables the expansion of racial capitalism through the accumulation and maintenance of wealth. Nuclear family formations are a direct historical process of colonization, conquest, and imperialism; this family formation is defined and understood as both the ideal and the only legitimate structure, both intimate and public, to uphold idealized individual morality, through property, ownership, and under the protection of US law [22]. Only capable through white heteronormative reproduction, the white nuclear family structure enables systems of capture, slavery, dispossession, accumulation, and other epistemic and physical violence to occur [22].

The racial categorization necessary for enslavement, coupled with the need for additional (re)production for the economic increase of slave owners, facilitated a eugenic logic wherein family was weaponized against enslaved people for capital gains. Hortense Spillers describes the ways in which race, gender, and the family serve as an ideological analytic tool utilized during colonialism and the transatlantic slave trade, and how enslaved African peoples were understood as “kinless” and even “genderless” in order to justify violent dispossession and enslavement [21]. The family here is a category only available to white European settlers, to ensure that property relations and capital are bound by the biological reproduction of whiteness.

The category of family was defined—and redefined—for slave owners. Longstanding norms and laws of patrilineal heredity and inheritance were shifted to ensure that a child born of an enslaved Black woman and a free white man would be enslaved, remaining *as* property rather than being entitled *to* property [23]. Black children were not the kin of their parents but rather the property of white slave owners. Enslaved people were coupled for reproductive increase and profit; these family formations were forced on people but not recognized or respected as an autonomous structure or system. Any biological reproduction by enslaved Africans during this time was owned and controlled by white settlers to ensure their “stock” of enslaved labor would flourish, enabling their profit to increase [24]. Family formations were coerced for capital incentives but not recognized, honored, or protected under colonial rule or law. Once a group becomes property, or are similarly devalued as expressed in the quote above, they become a tool for the capital gain of the state, and their rights to reproduction, family, and culture are diminished or eliminated completely.

In tandem to the transatlantic slave trade, the colonization of the so-called Americas also laid foundational ideologies of family structure that were pivotal to the process of colonization. The same principles of racial capitalism and eugenic ideology, including a hierarchical and violent ordering structure, were seen, both broadly in the treatment of Indigenous peoples and specifically in the way the family was controlled to dissolve communal economic principles and create new laborers within the white, dominant capitalist system [22]. This can be seen across numerous US policies over hundreds of years. Under the General Allotment Act of 1887, Indigenous lands were broken up and issued out as parcels based both on the value of the land as well as “members’ marital and dependent status” [16], delegitimizing and dismantling Indigenous family formations for the benefit of white settler capitalist accumulation. Residential Boarding Schools were established across the US and Canada with a goal of eliminating traditional Indigenous ways of life, language, and family structures and attempting to assimilate Indigenous children into white-American culture. Children were separated from their families, often for years at a time, forced to cut their hair and change their dress, punished for speaking their language, and forbidden from practicing non-Christian religions [25]. This eugenic practice of eliminating cultural traditions centered on the family, where Indigenous families were devalued, separated, and redefined for the purposes of maintaining the settler colonialist ideals.

Eugenics and control of the family through laws and policies have been continual features of US racial capitalism. For example, the centrality of the nuclear family in racial capitalism can be seen throughout various anti-immigrant laws, policies, and discourses in the 19th, 20th, and 21st centuries. The category of “Asian”, for example, was constructed and developed to allow the US courts to determine that immigrants from Asian countries were “racially distinct, inherently foreign, permanently unassimilable, and undesirable” [26]. California’s 19th century anti-miscegenation laws forbid marriages between whites and “Mongolians”, as Asian men were perceived as “threats” to white women [26]. The Cable Act of 1922 changed citizenship rights through mandating that US-born women who married Asian men (who were ineligible for citizenship) would lose their own US citizenship. Non-white immigrant families were—and continue to be—viewed as a threat to the white national identity of the US. We see this same rhetoric in discourses of “anchor babies” and the white nationalist panic surrounding declining white birth rates [26]. America has continued to fear not only the existence of immigrant families (first Asian and now Latinx and Muslim) in the US, “but also the creation of families with large numbers of non-white children” [26].

We have seen centuries of policies that limited the movement and reproduction of immigrant families in the name of upholding the white normative nuclear family structure. These policies and practices have continued to be reinvented for different populations and across different time periods, but the central features of the creation of hierarchical social categorization and othering, followed by the control of family and reproduction, have remained constant over time. These are the foundational threads of eugenic logic and racial capitalism and, while they have evolved, have persisted and remained constant in intent.

## 4. Prisons as Eugenic Institutions, Created by Racial Capitalism

“You’re already in state custody. Basically you’re a society throw away, so we don’t care. And this is what we’re going to do: We’re going to prevent women from the ability to get out–when they do get out–and have babies. We’re just going to handle this ourselves so we don’t end up creating a further pipeline from infancy straight to prison.”

—Formerly Incarcerated Survivor

When chattel slavery ended, there was a need to maintain a system of racialized hierarchical differentiation to extend discipline and control over the bodies and lives of people who had been enslaved. While the demands of capitalist accumulation remained, the legality and morality of blatant discrimination based on racial categories became more fraught. Reformed mechanisms for dispossession and hierarchical ordering were required. This occurred first via the Black codes, laws that specifically targeted Black individuals and group movements, developing into Jim Crow policies, and then, after the Civil Rights Movement of the 1960s instituted new protections and rights, via the criminal legal system [27].

When the 13th Amendment was ratified in 1865, it abolished slavery but left one clear exception: “except as punishment for crime whereof the party shall have been duly convicted” [28]. This exception left an opening to continue slavery and involuntary servitude and opened a possibility for the reification of similar modes of social ordering and categorical othering through the construction of criminal behavior specifically targeting certain populations. As John Ehrlichman, an aid to Richard Nixon, admitted in 1994,

You want to know what this [war on drugs] was really all about? The Nixon campaign in 1968, and the Nixon White House after that, had two enemies: the antiwar left and black people. You understand what I’m saying? We knew we couldn’t make it illegal to be either against the war or black, but by getting the public to associate the hippies with marijuana and blacks with heroin, and then criminalizing both heavily, we could disrupt those communities. We could arrest their leaders, raid their homes, break up their meetings, and vilify them night after night on the evening news [29].

This describes the work of creating and sustaining categories of differentiation and otherness. Approximately 352,000 people were incarcerated in the US in 1970, early in Nixon’s administration; by 2010, that number had risen to 2.28 million [30]. After 50 years of policies and the explicit creation of new categories of deviance under a racial capitalist regime, “the prison system under capitalism is overwhelmingly a repressive institution, an appendage of its state apparatus employed to maintain exploitative and oppressive social conditions” [31].

The criminal legal system, like chattel slavery, South African apartheid, segregation, immigration policies, the institutionalization of “disabled” and “deviant” people, and countless other examples, is both predicated on and creates categories of difference and hierarchical structures, which enact biopolitical control of populations through reproduction, based in racism and enforced through state mechanisms of power and violence [32]. These constructed categories feel natural and automatic. They are not concrete, stable, individual characteristics. Rather, the categories shift based on context and social and culture changes [33]. The construction of these categories is meant to feel like the natural societal order. The criminal legal system relies on this. In order to incarcerate people—much like in order to enslave people—we (both the captors and the broader society who allows it) must undergo a fundamental transformation in our understanding of these people and our relationship to them. We cannot see them as people like us; they must be transformed into an *other*.

This othering process is one of the key forms of work done by prisons. Prisons allow us to maintain the system of racial capitalism through the creation and sustainment of a system of hierarchical differentiation, justified through a constructed notion of criminality. As Ruth Wilson Gilmore described, racism makes it possible “to become so detached from another human being that another person with a different skin color might not even seem human” [34]. The criminal legal system has extended and legitimized this detachment through the creation of the “criminal” as a violent other who is morally corrupt and in need of containment. The incarceration of the “criminal” reinforces itself: one who is dangerous must be incarcerated; therefore, one who is incarcerated must be dangerous. The criminal is a bad person, distant and distinct from us. They are one deserving of punishment and the loss of rights. They must be controlled for our protection, benefit and, ultimately, economic increase. Yet, crime is socially constructed. A crime is a violation of the law, and “laws change, depending on what, in a social order, counts as stability, and who, in a social order, needs to be controlled” [35]. This allows for the criminalization of an evolving “other”, to suit the needs of the racial capitalist order. Yet, this work has been invisibilized, and the response to alleged criminality is normalized as the natural order.

When a person is convicted of a crime—or even stopped by police, detained, or arrested—their status as criminal or presumed criminal is deserving of punishment and control, often starting with the loss of rights and autonomy [36]. When we accept the existence of “criminals” as a class of being who need and deserve punishment, and from whom we need to be protected, it is reasonable to think that this is a group that should not have the privileges and responsibility of a family. When the institution of the family is viewed as being for the good of the larger society, for the benefit of the white middle class, the criminal is naturally viewed as both a threat to the structure of family and undeserving of a family of one’s own.

This has, in many ways, been codified into law. In 2002, a man named William Gerber lost an appeal in the 9th Circuit Court of Appeals for his case against Mule Creek State Prison in California. Gerber had asked to be able to send sperm to his wife so that she could become pregnant via artificial insemination. Mr. Gerber and his wife were both in their 40s, and he was serving a life sentence. Time was running out for them to be able to create a biological family. The court ruled that “the right to procreate is fundamentally inconsistent with incarceration” [37]. The court went on to draw a distinction between the right to be free from forced surgical sterilization, established under *Oklahoma v. Skinner*, and this request, noting “The right to procreate while incarcerated and the right to be free from surgical sterilization by prison officials are two very different things.” Presumably, the circuit judges presiding over this case were unaware that forced sterilizations were likely occurring in prisons in their state as they wrote this opinion.

The justices in this ruling held prior rulings that people who are incarcerated have a right to “maintain their procreative abilities for later use”, specifically emphasizing *later* and not present. The ruling noted that sterilization is “intrusive, permanent, and irreparable”. And yet, so too can be the loss of fertility that accompanies a long incarceration or the broken bonds of families violently ripped apart through incarceration.

While the Gerber case was brought by a man, family and reproductive control have always been key features of women’s incarceration in particular. Sarah Haley writes of the rise of female incarceration in the Jim Crow South and describes Black women being incarcerated due to the threat of their “unruly carnal impulses and perverse mothering [which] reproduced a class of black and mulatto male criminals that threatened the sanctity of the household and the safety of white women” [38]. Women in this era routinely served time in what had been men’s prisons and worked alongside men on chain gangs in labor camps. Haley asserts these institutions served to both punish Black women for their deviance and extract their free labor, while controlling their reproductive capacity [38]. Incarceration during this time was not only a tangible tool of discipline and management but an ideological apparatus that enabled the further reproduction of a gendered racial capitalism.

## 5. The Reproductive (In)Justice of Prisons

“Prison totally decimates family.”

—Formerly Incarcerated Survivor

When the forced sterilizations in California prisons came to light in 2013, the issue sparked a firestorm of news, commentaries, discussions, and advocacy. New legislation was passed the following year in California, outlawing tubal ligations for anyone incarcerated in the state. And yet, these tubal ligations were only the tip of the iceberg. A state audit revealed that between 2005 and 2012 [39], nearly 800 patients incarcerated in California’s women’s prisons underwent sterilizing procedures, including hysterectomies, oophorectomies, and uterine ablations. While these procedures are common and often medically necessary or emergent, many patients described being forced or coerced to undergo these procedures without proper informed consent, without their knowledge, or under devious means [32]. Importantly, many of these individuals describe feeling and experiencing the same eugenic logic expressed by James Heinrich. We will likely never know how many of these patients received care under duress or without proper medical rationale.

And yet, the experiences of these 800 individuals are still just one aspect of the eugenic nature of prisons. First, there are the countless others who may have been sterilized from 1979, when the practice of compulsory sterilization in state institutions ended in California, to the present day. We know from reports produced from the California Correctional Healthcare Services (2020) that as recently as 2020, a patient who was incarcerated in a state prison underwent a tubal ligation. Many patients, typically at least 10 per year, continue to undergo other sterilization procedures, with no way of knowing if these procedures happened after true informed consent [40].

But eugenics is larger than sterilization procedures. As we have described, the eugenic logic embedded in racial capitalism has not been limited to the parameters of the body. In the same way that violent dispossession of family and community were central to slavery, prisons enact eugenic logics through the disruption of family and communities. Eugenics is not limited to the violation of the right to have children but includes the overall enforcement and policing of reproduction and family.

Over the last decade, the state of California has made efforts, at least through legislation, to maintain the *bodily* right to have children. Yet, as we examine incarceration from a reproductive justice framework, it is clear the state has not intervened in meaningful ways to uphold the tenets of reproductive justice. Reproductive Justice (RJ) is a framework developed by Black Feminists and speaks to the limitations of the reproductive rights movement, which often centers the needs of white, middle-class, cis women [41]. Sister Song, a leading reproductive justice organization, defines RJ as the “human right to maintain personal bodily autonomy, have children, not have children, and parent the children we have in safe and sustainable communities” [42]. Forced and coerced sterilization necessarily disrupts personal bodily autonomy and the right to have children. Prisons violently disrupt all four tenets.

### 5.1. Personal Bodily Autonomy

While protections for medical informed consent exist, at least in the law, for people who are incarcerated, the right to personal bodily autonomy is fundamentally disrupted and challenged by incarceration. When incarcerated, one’s body becomes the property of the state. Individuals lose the right to determine what they eat, where and when they sleep, and how they spend their time. Even in medical care, where certain rights (e.g., the right to refuse medical care) are protected, threats of harassment and retaliation remain, and the right to seek care remains conditional on the structural and punitive conditions of incarceration.

### 5.2. Right to Have Children

The right to have children is the most visible and visceral when taken away by forced sterilization, yet the carceral system disrupts this right in many ways. When we incarcerate people, especially people assigned female at birth, for decades-long sentences, we create a near impossibility of planned family formation. Prisons serve to eliminate the ability to have children *by design*. This is not an unfortunate or unintended consequence of incarceration but rather a central feature of how the system is designed. The length of sentences we see in the US extend beyond any other industrialized nation. These sentences, including the one in seven incarcerated people serving life sentences [43], have not been shown to be deterrents for crimes nor to make our society safer [44]. As Oleson posited, “If a three-strikes law does not increase deterrence, and is financially unsustainable, there must be some justification for its enactment. A eugenics style policy might be one explanation. A prison sentence of 25 years to life would generally mean that if the person is released, he would no longer be biologically able to have children” [45]. Viewing eugenics as an intentional feature of prisons and sentencing puts into stark relief the racial bias in sentencing and the over-policing of Black and Brown communities.

### 5.3. Right to Not Have Children

Prisons and jails also disrupt the right *not* to have children. In 2022, the US Supreme Court overturned Roe v. Wade, further eroding the right to an abortion in the US. While already not fully protected or guaranteed for many, this decision put a further spotlight on the potential for the criminalization of bodies that do not, cannot, or will not carry babies to term. In the same decades in which people in California prisons were undergoing forced sterilizations, others in California were incarcerated for miscarriages and stillbirths that were suspected to be caused by drug use [46,47]. This will only grow across the country, as more and more states criminalize abortions earlier and earlier in pregnancy. The 2022 *Dobbs* decision that overturned *Roe* also made access to abortion for those incarcerated even more fraught. Even in states with a protected right to abortion, incarcerated patients may face numerous structural and interpersonal barriers to accessing abortion and other types of reproductive care while incarcerated [48,49]. Prisons and jails often deny access to birth control [50,51]. These policies often ignore the many reasons for birth control beyond the prevention of pregnancy and make it more challenging for people to avoid pregnancy, if desired, during and after incarceration.

### 5.4. The Right to Raise Children in Safe and Sustainable Communities

Finally, a core tenet of reproductive justice is the right to raise children in safe and sustainable communities. Prisons function as a violent disruption of family systems—terrorizing communities through policing as well as caging and separating children from loved ones. When people are incarcerated, they are unable to raise the children they already have or give birth to while incarcerated. While some people have family who can care for their children, and others may only have children in foster care for a short period, in many states parental rights are automatically terminated after children have been in the foster care system for a certain amount of time [11]. Children may be adopted by other families or languish in the foster care system regardless of the reason or length of incarceration. Prisons do not facilitate family reunification and continued parenting. In fact, they often do the opposite, with many individuals incarcerated long distances from home, and more prisons moving to video visits in place of in-person visits and charging exorbitant fees for electronic correspondence.

The absence of one parent due to police involvement or incarceration puts further surveillance on families through the often-mandated involvement of child protective services, making them more vulnerable to state intervention and family policing [11]. Desires to disrupt the deleterious cycle of incarceration often focus on the individuals who have been targeted by this system rather than the system itself. This leads to the eugenic logic embedded in this system: people who will be, are, or have been incarcerated should not have children because those children will then become a further burden on the state and likely become criminal or deviant themselves.

Reproductive oppression is not an unintended consequence of incarceration. It is a central and deliberate feature [52]. Incarceration controls families and reproduction in the name of public safety, based on categories of othering and difference. Prisons work to maintain, contain, and tear apart family formations that do not fit into the ideal nuclear biological family. Prisons—even in the absence of forced sterilization—serve as a form of structural eugenics, targeting Black, Brown, poor, queer, trans, and disabled bodies, all of whom are vastly overrepresented in the prison system [30] and all of whom have had their right to family and their family formations questioned for hundreds of years. Forced sterilizations have taken away the right to reproduce from some. Prisons, by design, intend to take away the ability to create, maintain, and raise families for all.

## 6. Discussion

“Incarceration is a tool of reproductive suppression. And the only way around that is abolition.”

—Aminah Elster [53]

In 2017, a Tennessee judge was reprimanded for offering reduced jail time in exchange for sterilization during sentencing decisions in his court [54]. In 2020, a whistleblower named Dawn Wooten came forward about forced sterilizations that were occurring at the Irwin Detention Center in Georgia [55]. In 2022, lawmakers in West Virginia considered a bill to lower prison time for people convicted for drug-related offenses who agreed to sterilization. When asked about this policy, one lawmaker expressed a desire to not “bring any more drug babies into the system”, arguing that “until we cut the head of the snake off…we’re trying to take care of the problem after the fact” [56]. What may have seemed like shocking or extreme rhetoric or actions that took place in California in the early 2000s were in fact the continuation of a longstanding pattern of othering, eugenics, and family and reproductive control in the United States.

When we began research on the forced sterilizations in California prisons, our intent was to understand how and why a prison system was conducting eugenic sterilizations in the 21st century. We wanted to document the stories of the survivors and identify tools and resources to guard against these types of practices. We came to see that this was too narrow of a view. Through conversations with advocates, organizers, scholars, and survivors, we have come to understand the ways in which prisons were always, already eugenic institutions. With this paper, our goal was to demonstrate the many ways in which prisons, as an institution created by racial capitalism, enact eugenics as a method of familial and reproductive control.

Too often, eugenics is seen as synonymous with compulsory sterilizations. While this has been a feature of eugenics in prisons, it is not the only example of the enactment of eugenic logic. When considering not just a single-issue rights-based framework—such as the right to have children—but a *reproductive justice*-based framework, we see the numerous ways that the US system of incarceration, and the carceral and eugenic logics embedded therein, has controlled and oppressed family and reproduction beyond just the physical ability to reproduce. A reproductive justice-based framework allows us to understand the role of the family in the creation of carceral logics and to interrogate how and why we accept the disruption and dissolution of families in the name of public safety and social control.

While most US-based compulsory eugenics programs officially ended in the 20th century, eugenic logic and practice have been socially and materially reproduced by the state through prisons, which serve as a foundational mechanism to extend the function of racial capitalism. We cannot and will not end eugenics by ending forced sterilizations. As one of our key collaborators in this work said, “Incarceration is a tool of reproductive suppression. And the only way around that is abolition” [53]. When we consider eugenics, considering only surgical and scientific eugenics is insufficient. *Social and structural eugenics* must also be centered. By limiting reproductive capacity and potential and inhibiting the future potential of family building and self-determination of already marginal and vulnerable groups, the state has continued a legacy of violent family separation and state control over deviant reproductive bodies. The only way to eradicate the eugenic logic embedded in prisons is to dismantle the institutions, and the racial capitalism, that have created the othering that has allowed for eugenics to be perpetuated for the last four hundred years.

The forced and coerced sterilizations of people incarcerated in California women’s prisons is a confronting example of eugenics—women under state control being forcibly sterilized based on socially constructed categories of otherness and risk. However, the insidious nature of eugenic logic and the examples of reproductive oppression extend even beyond the abusive surgical interventions performed on women like Moonbeam. Prisons, by design, control bodies and autonomy [52]. Prisons, by design, control, limit and define families. Racial capitalism creates and necessitates social ordering, and the state uses that ordering to organize reproduction and families based in eugenic logic. Prisons are an extension of that project. Prisons are eugenic institutions.

## Data Availability

The data presented in this article are not readily available due to privacy and ethical restrictions.
